# Searching in CCTV: effects of organisation in the multiplex

**DOI:** 10.1186/s41235-021-00277-2

**Published:** 2021-02-18

**Authors:** Benjamin W. Tatler

**Affiliations:** School of Psychology, University of Aberdeen, Aberdeen, AB24 3FX Scotland, UK

**Keywords:** Visual search, CCTV, RT, Semantics, Multiplex, Scenes

## Abstract

CCTV plays a prominent role in public security, health and safety. Monitoring large arrays of CCTV camera feeds is a visually and cognitively demanding task. Arranging the scenes by geographical proximity in the surveilled environment has been recommended to reduce this demand, but empirical tests of this method have failed to find any benefit. The present study tests an alternative method for arranging scenes, based on psychological principles from literature on visual search and scene perception: grouping scenes by semantic similarity. Searching for a particular scene in the array—a common task in reactive and proactive surveillance—was faster when scenes were arranged by semantic category. This effect was found only when scenes were separated by gaps for participants who were not made aware that scenes in the multiplex were grouped by semantics (Experiment 1), but irrespective of whether scenes were separated by gaps or not for participants who were made aware of this grouping (Experiment 2). When target frequency varied between scene categories—mirroring unequal distributions of crime over space—the benefit of organising scenes by semantic category was enhanced for scenes in the most frequently searched-for category, without any statistical evidence for a cost when searching for rarely searched-for categories (Experiment 3). The findings extend current understanding of the role of within-scene semantics in visual search, to encompass between-scene semantic relationships. Furthermore, the findings suggest that arranging scenes in the CCTV control room by semantic category is likely to assist operators in finding specific scenes during surveillance.

In many societies, CCTV surveillance has come to play a prominent role in public security, health and safety, providing a deterrent for criminal activities (Piza et al. 2019), a retrospective record of video evidence for review after an event (Piza et al. 2019) and, crucially, online monitoring of public spaces. Online monitoring with CCTV can be proactive or reactive (Keval and Sasse 2010). Proactive surveillance involves active monitoring of the CCTV array by operators in order to detect crime. For example, operators proactively monitor for suspicious activity (Gelernter 2013; Howard et al. 2013; Troscianko et al. 2004). Reactive surveillance involves operators responding to requests from other individuals within the control room or elsewhere. For example, police radio the CCTV control room to request information about a suspect, location or developing incident (Keval and Sasse 2010). Both proactive and reactive surveillance can aid early detection of crimes and, thus, avoid minor incidents escalating by allowing rapid and effective deployment of police resources (Levesley and Martin 2005; Piza et al. 2015, 2017).

In order to maximise simultaneous access to visual information for these surveillance tasks, most CCTV control rooms show data from multiple cameras simultaneously on a “data wall” which displays a multiplex of camera feeds (for examples, see Fig. 1). Monitoring multiplexed arrays of CCTV camera feeds in the control room is a visually and cognitively demanding task for the operator, so any way in which the operator can be supported in this task will have clear public safety benefits. For a thorough discussion of the cognitive demands of CCTV monitoring see Hodgetts et al. (2017). In order to aid the operators in this task, recommendations have been made for configuring the array of camera feeds in the data wall to reflect the underlying geography of the surveilled environment (Donald 1998; Pikaar et al. 2015; Wallace and Diffley 1998), and some CCTV control rooms use this organising principle. However, empirical tests of this method of arranging scenes have failed to show any benefit (Harris et al. 2008; Stedmon et al. 2011). The present study applies theoretical understanding from visual search and scene perception to propose and evaluate an alternative method for arranging camera feeds in the control room: grouping scenes by semantic similarity. The present study also introduces a novel paradigm for CCTV research. Previous studies have focussed on detecting flashpoint aggression (Troscianko et al. 2004), suspicious behaviour (Howard et al. 2013) and following suspects (Harris et al. 2008; Stedmon et al. 2011)—or a mixture of such incidents (Hodgetts et al. 2018)—and have used relatively small multiplexes of four (Howard et al. 2009, 2013), six (Harris et al. 2008; Hodgetts et al. 2018; Stedmon et al. 2011), nine (Stainer et al. 2017) or 16 (Tickner and Poulton 1973) scenes. The present study employed a task that simulates a common reactive surveillance task in the control room—finding a target scene (Keval and Sasse 2010)—and used a multiplex of 27 scenes taken from police CCTV surveillance cameras in one Scottish city.

**Fig. 1:**
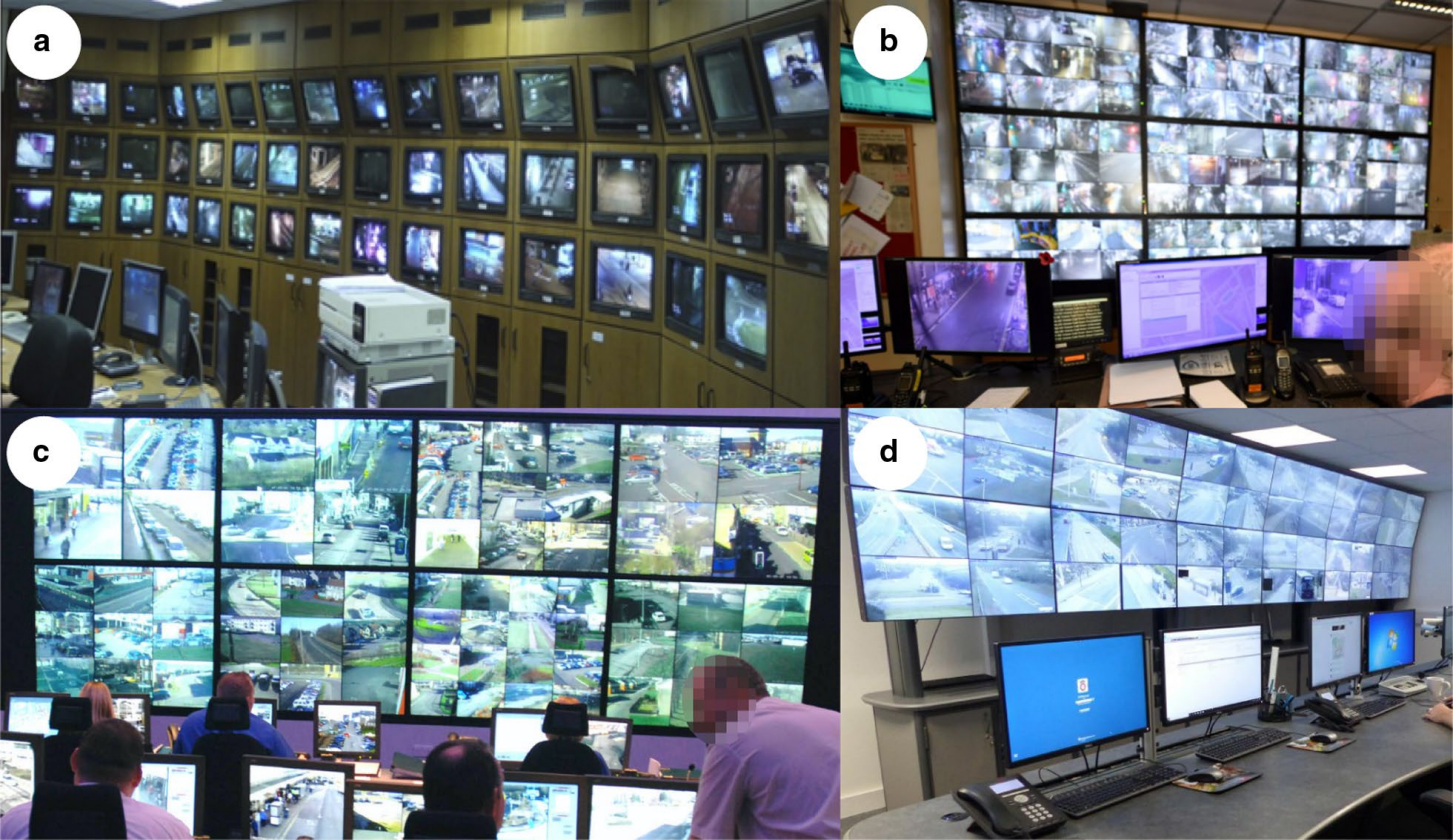
CCTV control rooms. **a** A control room with separate monitors for each scene, separated by the frames of each monitor. **b**–**d** Control rooms with several large screens, each showing multiple camera feeds. Within the large monitors, individual feeds have no separation between them. The number of scenes per monitor varies considerably between the three examples shown: in **d** there are four camera feeds on every large monitor; in **b** there are 12 camera feeds per monitor and in **c** the number varies from 4 to 16 between monitors. Image sources: **a** Photograph taken by Kenneth C. Scott-Brown for Stainer, Scott-Brown and Tatler (2013), **b**–**d** publically available images downloaded from Google image search

*Why organising the multiplex might help CCTV operators*

Based on previous studies of detection performance (Tickner and Poulton 1973) and operator confidence in detection abilities (Wallace et al. 1997) when monitoring multiple scenes simultaneously, guidelines have been proposed that a single operator should be required to monitor no more than 16 camera feeds simultaneously, and that this should be reduced to nine feeds if they show considerable movement (Schreibers et al. 2012; Wood and Clarke 2006).

In practical terms, having a 16:1 camera:operator ratio in the CCTV control room may be difficult to achieve, particularly in large cities that have thousands of cameras to be monitored. In practice, operators are often required to monitor far more than this: Gill and Spriggs (2005) studied 14 CCTV control rooms and found higher camera:operator ratios in all cases. Indeed, the minimum number of cameras per operator was 20, with up to 175 cameras displayed simultaneously on the data wall (Gill and Spriggs 2005; Gill et al. 2005).

Given this gap between recommendations and what is likely to be practically achievable, an alternative approach is to consider whether the data wall can be configured in ways that aid the operator’s ability to monitor the scenes. It has been recommended, for example, that grouping scenes using logical organising principles in the data wall might help operators (Donald 1998; International Organization for Standardization 2013; Pikaar et al. 2015; Wallace and Diffley 1998).

Until relatively recently, reorganising the arrangement of the data wall in a CCTV control room was not straightforward. This is because data walls in CCTV control rooms were created using banks of individual monitors, each receiving input from one or several cameras in the surveilled environment (Fig. 1a). A consequence of systems with hard-wiring between cameras and display screens is that the arrangement of camera feeds on a data wall can be unsystematic, with feeds from newly installed cameras being mapped to where there is space to add a new display screen in the data wall (Harris et al. 2008; Stainer et al. 2013). Operators themselves report that one of the difficulties in use of the data wall of displays is that it can be hard to search through and find particular scenes due to the ad hoc arrangement of displays in the data wall (Keval and Sasse 2010).

In modern control rooms, however, data walls are typically made up of a small number of large monitors, with software solutions to divide up each large monitor to show feeds from a large number of cameras (Fig. 1b–d). Because the mapping of camera feed to display screens in current systems is software-controlled, the arrangement of feeds on the data wall can be configured (and re-configured) easily to reflect recommended organising principles.

*Organising the multiplex by geographical proximity*

There are obvious potential benefits of geographical organisation of a CCTV data wall when following a suspect—an important and common task in CCTV surveillance (Goold 2004)—or needing to check areas near to a currently monitored camera feed as these would then be nearby in the multiplex. Furthermore, one of the reasons that grouping scenes within a multiplex may support the operators in the CCTV control room is that crime is not uniformly distributed over space and time. Rather, there are particular locations where crime is more likely than others (for historical reviews see Chainey and Ratcliffe 2013; Weisburd et al. 2009), with studies showing that cities have specific crime “hot spots” (Sherman 1995; Sherman et al. 1989). For example, Sherman et al. (1989) found that 3% of the addresses in Minneapolis, USA, accounted for 50% of the calls that police responded to. Others have found similarly concentrated clusters of crime in cities, with a small number of locations accounting for a large proportion of crime (Weisburd et al. 2004, 2009). Moreover, where crime is likely to occur depends on the type of crime (Chainey and Ratcliffe 2013; Gupta et al. 2012). Crime rates vary not only over space but also over time (Ratcliffe 2010): robberies are more common in the evening (Felson and Poulsen 2003), and theft of vehicles tends to be from non-residential areas during the day, but residential areas during the night (Ratcliffe 2002).

A result of the non-uniform distribution of crime in space and time is that at any one moment in the CCTV control room, certain camera feeds will be of greater priority for proactive monitoring than others, and are more likely to be the target of reactive surveillance, when a radio call for information about an incident or location is made to the CCTV operators. Placing these high-crime-risk scenes together in the CCTV multiplex is likely to be of benefit for the operators. The dynamic nature of crime patterns over space and time throughout the day and night means that the high-crime-risk scenes will vary over a 24-h period and therefore the best grouping of scenes within the multiplex may also vary across operator shifts. Indeed, police CCTV operators preferentially monitor a subset of scenes within the multiplex and the subsets differ at night from those during the day (Stainer et al. 2013), and novice participants spontaneously adopt viewing strategies of prioritising certain scenes during surveillance tasks (Hodgetts et al. 2018).

Despite recommendations that it will aid operators (Donald 1998; International Organization for Standardization 2013; Pikaar et al. 2015; Wallace and Diffley 1998), and the use of this organising principle in some CCTV control rooms (K. C. Scott-Brown, personal communication, July 2, 2020), empirical investigations have failed to find beneficial effects of arranging displays according to logical, geographical relationships on accuracy or reaction times for tracking suspects across displays or in detecting suspicious behaviour (Harris et al. 2008; Stedmon et al. 2011).

*Organising the multiplex by scene semantics*

In the present study an alternative way of organising scenes in the multiplex is evaluated: grouping camera feeds by what type of scene they show (i.e. by the semantic category of the scene). Changes in the types of crime that are most common over the day will result in particular categories of scenes being more commonly the target of proactive and reactive surveillance over the 24-h period. These categories may not comprise scenes that are geographically clustered: for example, non-residential car parks will be the most common locations for car theft during the day (Felson and Poulsen 2003), yet these car parks will be distributed over the city, so grouping by semantics rather than geography will place these high-risk camera feeds together in the multiplex. Grouping scenes in this way satisfies previous recommendations of logical grouping in the multiplex (Pikaar et al. 2015) but does so on the basis of an alternative organising principle, informed by what is known about the associations between particular types of crime and particular types of scene (Ratcliffe 2010). The possibility of grouping scenes by semantic category in the CCTV control room has not been tested empirically, but is used in some CCTV control centres; for example, in Manchester, because National Car Parks and Manchester Police share a common control room, all car park camera feeds are grouped together on the data wall irrespective of their location in the city (K. C. Scott-Brown, personal communication, July 2, 2020).

From a theoretical perspective, there are grounds to suggest that grouping scenes by semantic content is likely to help the operator find a particular scene efficiently, as is required in reactive surveillance. Semantic understanding of a scene is rapid and plays an important role in guiding how people search within it. The semantic category, or gist, of a scene can be extracted within the first 125 ms of a scene appearing (Biederman 1972; Potter 1975, 1976), or less (Fabre-Thorpe 2011). Spatial organisation within a scene is similarly rapidly extracted and used to guide search behaviour. When searching for an object within a scene, the first few fixations are directed to the region within the scene in which the object is expected (Ehinger et al. 2009; Torralba et al. 2006) and the first saccade after scene onset will often be directed to the expected location of a target object even when the target is not there, provided that there is a distractor object within that region to select (Spotorno et al. 2014). Beyond the effects of these learnt associations between objects and scene regions, scene categorisation can guide search on-the-fly for novel object-context relationships. If participants are told where an object is likely to occur, they will constrain their search to these likely regions even when they have no prior knowledge to base this on (Zelinsky and Schmidt 2009). How semantic understanding of scenes might help guide search in arrays of multiple scenes is less well understood. To guide search to a specific scene within a multiplex of scenes, rapid semantic categorisation must be possible in peripheral vision and for multiple scenes in parallel. Indeed, Potter and Fox (2009) and Rousselet et al. (2004) concluded that, despite some performance costs as the number of scenes increased, up to four scenes presented simultaneously in peripheral vision can be rapidly categorised in parallel. Conversely, Greene and Wolfe (2011) found inefficient search when up to four scenes were presented simultaneously, suggesting that processing global scene properties was impaired in multiple-scene search. Furthermore, when presenting up to 16 scenes simultaneously, VanRullen et al. (2004) concluded that the extent of performance costs with increasing scene number suggested serial processing of scene’s gist, but this serial processing was conducted in peripheral vision at around 40 ms per item and did not require each scene to be looked at. Thus, whether parallel or serial, and whether efficient or inefficient, gists from multiple simultaneously presented scenes can be extracted in peripheral vision, prior to actually looking at the scene.

While the above evidence suggests that peripheral extraction of semantic categories from scenes in the multiplex may help to guide the process of searching for a particular scene, it should be noted that these prior studies have used only a small number of scenes compared to a typical CCTV data wall. Furthermore, while VanRullen et al. (2004) used up to 16 scenes, they found that performance was at chance when there were more than eight scenes presented at once. Peripheral categorisation of individual scenes, therefore, is likely to be of limited use within the large number of scenes typically presented in a CCTV multiplex. It may, however, be possible to scaffold this semantic search, providing sections of the multiplex that have common semantic category. Once the locations of each cluster of semantically similar scenes are learnt, search may be guided to the relevant region of the array based on the semantic category of the searched-for scene. In this way the CCTV array of scenes might itself act as an overall “scene” with regions of common semantics within groups of camera feeds. Search could then potentially rapidly be constrained to the subset of camera feeds in the array that have the same semantic category as the searched-for scene.

Difficulties for peripherally processing scenes in larger arrays are likely to stem from at least two sources: how far a scene is in peripheral vision and the difficulties associated with individuating a scene when it is surrounded by other scenes. Even at eccentricities of up to 70$^{\circ }$, people are able to classify scenes according to their content (Boucart et al. 2016; Thorpe et al. 2001) and to identify the semantic category of a scene (Boucart et al. 2013). However, it is uncertain how the presence of surrounding scenes might influence this ability in far peripheral vision when viewing scenes in a multiplex. Object recognition is impaired when the object is surrounded by other objects (Levi 2008; Pelli and Tillman 2008; Whitney and Levi 2011) or placed within the larger context of a scene (Davenport and Potter 2004; Vanmarcke and Wagemans 2016).

The problem of distance to a scene in the multiplex is a necessary constraint when viewing the CCTV data wall, but the detrimental effects arising from surrounding scenes might be mitigated if individual scenes can be rendered as more visually distinct in peripheral vision. For example, introducing a gap between scenes might aid peripheral processing of scenes and, thus, support search for a particular scene. Previous work has shown that introducing gridlines within a scene to physically separate sections of scene content can be beneficial when scene context is important for the task (Varakin et al. 2013). In modern CCTV data walls, scenes typically abut with no physical separation between individual scenes (see Fig. 1b–d), whereas, in older control rooms, the use of one physical monitor per camera feed meant that scenes were separated by the bezel (or border) of each monitor in the multiplex (see Fig. 1a). It may, therefore, be that modern systems make the task of searching for particular scenes during reactive surveillance more difficult than it was in older control room settings.

*The present study*

In the present study the effects of two organising principles in the CCTV data wall on search for a target scene were considered: whether scenes are grouped by semantic category and whether individual scenes abut or are separated by gaps. Three experiments were conducted in order to characterise how these organising principles influence the time to find a target scene amongst an array of 27 static images of scenes captured from CCTV surveillance cameras in a single city in Scotland. These images were drawn from three distinct semantic categories.

By considering effects on search for a target scene, the present study most closely models a common task in reactive surveillance, essentially modelling the initial orienting to a particular camera feed after the operator is radioed and asked to report on a particular location. The task is also relevant to aspects of proactive search, particularly when monitoring for specific crimes in particular areas.

Specifically, the present study addresses the question of whether physical separation between scenes and organising scenes by semantic category influence search time for a particular scene when participants are not made aware of the arrangement (Experiment 1) and when they are informed where each category of scene will be displayed (Experiment 2). This allows evaluation of whether introducing gaps between scenes and organising scenes by semantic category is beneficial for search, and whether this depends upon explicit knowledge of the scene arrangement in the multiplex. In Experiment 3, the effect of organising the scenes in the multiplex by semantic category is considered in situations when the target frequency varies between scene categories. This experiment provides a way of evaluating the costs and benefits of organising scenes by semantic category when certain types of scenes are more likely to be searched for—as is often the case in reactive and proactive surveillance due variations in patterns of crime over space and time. Collectively, the experiments provide a first empirical test of the effects of physical separation and organisation by semantic category on search within a CCTV multiplex.

*The multiplex search paradigm*

The present study examined the impact of scene arrangement on the speed with which viewers orient to a particular scene in a multiplex scene array. The paradigm was developed to be relevant to this component of real-world surveillance, while offering the necessary experimental control and amount of data typical of laboratory-based studies.

Participants repeatedly searched a multiplex array of 27 static scenes, which, to reflect operators’ experience in the CCTV control room, had a fixed scene arrangement in each block of trials. A brief preview of a different and randomly selected target scene was presented on each trial, before the array. As there were no target-absent trials, to ensure that participants responded only after finding the target scene, they also had to discriminate between a small T or L in the top left corner of that scene.

The experimental task most closely models a common task in reactive surveillance, essentially modelling the initial orienting to a particular camera feed after the operator is radioed and asked to report on a particular location. The task is also relevant to aspects of proactive search, particularly when monitoring for specific crimes in particular areas. While the paradigm aims to provide a laboratory-deliverable model of the surveillance task of orienting to a target scene for subsequent monitoring, it differs in several ways from the real-world task in the control room.

First, the physical setup had a smaller screen. The UK government (Centre for the Protection of National Infrastructure 2016) recommends that each monitor in the control room has a width of 10$^{\circ }$–17$^{\circ }$, which means 2.5$^{\circ }$–8.5$^{\circ }$ for each individual scene. Here, individual scenes were around 3° wide and, thus, at the lower end of this range.

Second, CCTV control room multiplexes typically contain many more scene images (see Fig. 1). However, as those images are monitored by multiple operators, the 27 scenes in this study are likely to be within the range—albeit at the lower end—that each operator is required to monitor (Gill et al. 2005), offering a suitable insight into the demands placed on individual operators.

Third, a set of scene images captured from real-world CCTV cameras was used, with realistic contents and viewing angles. However, these image were static, while camera feeds in real control rooms show dynamically changing scenes, where motion cues may contribute to operators’ decisions about where to attend (Stainer et al. 2013), but also increase the task demands (Hodgetts et al. 2017).

Fourth, images were used as scene target templates, whereas operators in reactive surveillance are likely to be verbally asked to orient to a specific location, like a certain pub or shop. Search is usually less efficient and relies more on expectations with verbal templates than image templates (Spotorno et al. 2014), as images provide a more precise definition of the target (Malcolm and Henderson 2009). However, the surveilled scenes are well known to the expert operator, and a verbal description is, thus, likely to define the target rather precisely. The participants had no prior knowledge of the scenes, and so the precision of an image template may be appropriate as a model for the surveillance task carried out by expert operators. Furthermore, as an array of often-similar scenes (e.g. nine images of traffic scenes, see Fig. 1) was used, it would have been difficult to uniquely identify a scene via a verbal description.

Fifth and finally, in real-world surveillance, after orienting to the target scene, the operator will inspect it to monitor its content or report on activity within it. The T/L discrimination in the present paradigm was not intended to mimic these inspection processes, but it only served to ensure active search and attentional allocation to the target scene. An alternative would have been to ask participants to report some detail of the target scene’s content. However, given repeated presentation of the same images across trials, that task would be impractical.

Overall, this paradigm includes several elements that in part remove it from the real-world surveillance task it was designed to model. However, they were introduced to produce a laboratory-deliverable paradigm suitable for naive participants and for data collection across many trials. The core aim of the paradigm was to model the process of orienting to a particular scene within the multiplex, and the simplifications in terms of the search template and discrimination task were made to preserve this aspect of the surveillance task.

## Experiment 1

### Method

#### Design

The experiment followed a $2\times 2$ repeated-measures design with two independent variables: the presence of gaps (borders) between scenes (present or absent) and the organisation of scenes in the multiplex (mixed randomly or organised by semantic category). The dependent variable was manual response time.

#### Participants

Thirty-six participants took part in the experiment. All participants were undergraduate students and took part voluntarily or for course credit. Participants were recruited by word of mouth and through the SONA recruitment system. All participants had normal or corrected-to-normal vision. Participants were naive to the purposes of the study. The study was approved by the local Psychology ethics committee (PEC/3510/2016/9).

There were no comparable published studies or pilot data to calculate the required sample size for this design given the planned linear mixed model analyses. A conservative estimate of the required sample size to detect a $2\times 2$ interaction within an ANOVA, assuming a medium effect size of 0.25 and a medium correlation of within-subject measurements of $r = .3$, calculated using G*Power 3 (Faul et al. 2007), suggests a required sample size of 32. Given that the linear mixed models (LMMs) used in the present study offer more power than ANOVAs by considering all trials (rather than averaging across trials in ANOVA) and simultansously estimating variance due to subjects and items (González et al. 2014; Kliegl et al. 2011), 32 participants are likely to be an overestimate of the required sample size to obtain 80% power. Simulations were subsequently run on the collected data to estimate power in the linear mixed model, and these simulations from the data collected in Experiment 1 were used to inform a priori power calculations for Experiments 2 and 3 (for details of these simulations see Data Analysis below).

#### Materials

Twenty-seven full-colour static screen captures from CCTV surveillance cameras located in the city of Dundee in Scotland were selected from a pool of 85 supplied by Police Scotland taken from their CCTV cameras for use in the present study. Stimuli were selected to fall into three categories: (1) cameras in city centre areas, (2) cameras in suburban areas and (3) traffic scenes. The category labels were merely for the experimenter and were at no point used in the experiment or made available to the participants. Nine camera images were selected for each of these three categories.

**Fig. 2:**
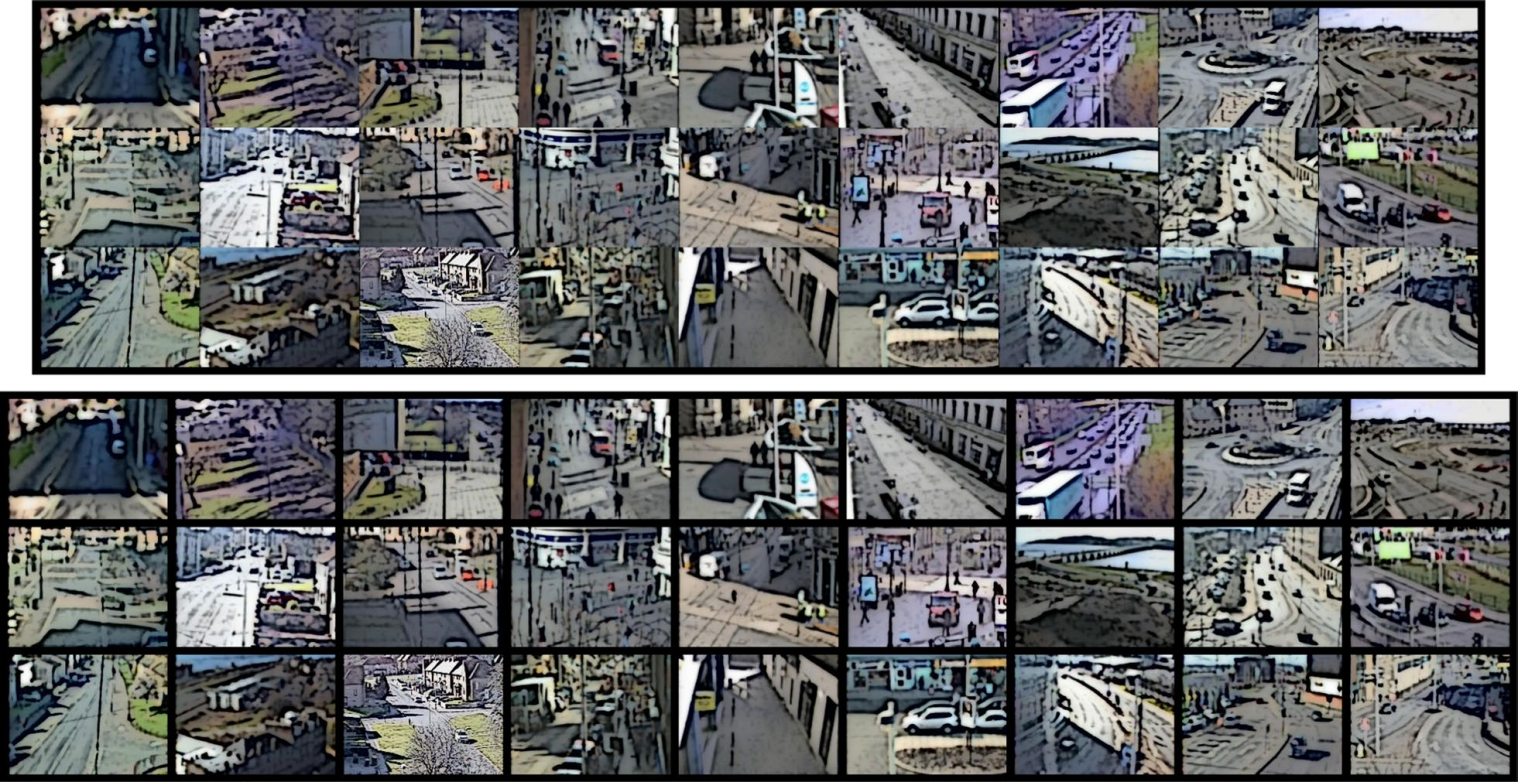
Example multiplexes. Examples are shown for a multiplex without borders between scenes (top) and for a multiplex with 10-pixel gaps between all scenes (bottom). Images have been modified for privacy reasons, but were shown as full colour photographic images. In these examples, scenes are shown arranged by category, with suburban scenes in the first three columns, city centre scenes in the central three columns and traffic scenes in the rightmost three columns

Images were re-sized to $200\times 150$ pixels for the present experiment and used to create a multiplex of nine scenes horizontally by 3 scenes vertically. The multiplex was created either with no gaps between scenes or with 10 pixel gaps to create borders between scenes depending on the experimental condition. Without borders, the resultant multiplex was $1800 \times 450$ pixels; with borders the multiplex was $1880 \times 470$ pixels. Figure 2 shows example multiplexes with and without borders between the scenes.

The experiment was run on a 2015 21.5” iMac connected to an LG smart TV monitor (Model 55SM5KB-BD) with a $1920 \times 1080$ pixel display area, mounted on the wall. The display area of the monitor measured 122 cm horizontally $\times 68\,$cm vertically. The bottom of the display area was 143 cm from the floor. Participants were seated approximately 260 cm from the display screen.

The experiment was built and run using SR Research Experiment Builder (v1.10.1630) software.

#### Procedure

The experiment was run in a dimly lit room with blinds covering the windows to avoid reflections on the screen. At the start of the experiment, information was provided about the nature of the task and the appearance of the multiplex, with an example multiplex shown during this instruction phase. Once the participant understood the task requirements, each participant carried out four blocks of 45 trials each. The four blocks corresponded to the four experimental conditions of the $2\times 2$ repeated-measures design, and the order of these blocks was randomised for each participant. Throughout the experiment instructions and stimuli were displayed on a black background, with instruction text appearing in white.

**Fig. 3:**
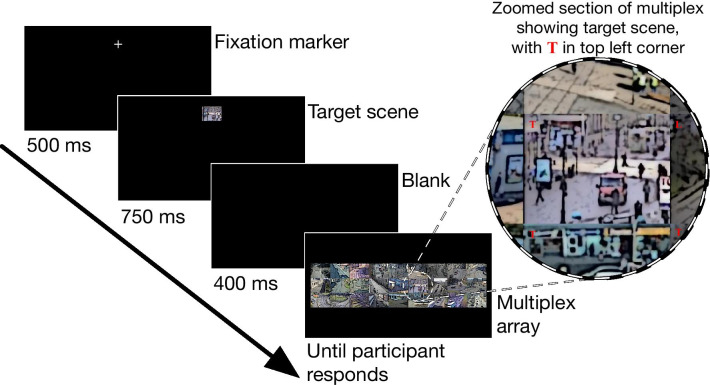
Experimental trial sequence. Screens and content are all drawn to scale, with the exception that the fixation marker here is drawn at a much-exaggerated size in order to make it visible at this scale. The insert shows an enlarged view of the target scene in the array; in this example there is a T in the top left corner. The participant’s task was to report whether the target scene contained a T or L

The task of the participant on each trial was to find a randomly selected scene within the multiplex array and report whether it had a small red letter T or L superimposed in its upper left corner. The trial sequence is shown in Fig. 3 . Each trial was self-initiated by the participant pressing the space bar when they were ready. This was immediately followed by a white fixation cross positioned at the horizontal centre of the screen, but above the location where the multiplex would subsequently appear (centred at pixel coordinates 920,186). The fixation cross was displayed for 500 ms before it was replaced by an image of one of the scenes from the multiplex that would act as the target scene for the current trial (with no letter in the top left corner), centred at the same location and displayed at $200 \times 150$ pixels. The target was selected randomly from the 27 experimental scenes at the start of each trial. The target remained visible for 750 ms. After a 400-ms blank screen, the multiplex search array was displayed and remained visible until the participant responded. Each scene in the multiplex had a small (font size 14, boldface Times New Roman) red T or L superimposed in its top left corner. The letter was small enough that it could not easily be seen in peripheral vision, requiring that the participant looked close to it in order to be able to report it accurately. Whether the target scene contained a T or L was determined randomly on every trial and 13 Ts and 13 Ls were randomly allocated to the 26 non-target scenes on every trial. Participants responded by pressing keys on the computer keyboard using the index fingers of each hand. The response keys were C and L, and these keys were covered with a blue and yellow sticker, respectively. Allocation of T and L to the blue and yellow stickers was counterbalanced across participants.

The purpose of the T/L discrimination was not to mimic any aspect of the real-world surveillance task, but rather to ensure that participants actively searched for and attended to the target scene on every trial. By randomly allocating Ts and Ls to each scene one each trial, participants had to search and attend to the target even if it had been the target of search on a previous trial.

The arrangement of scenes in the multiplex was fixed throughout each block—such that each scene appeared in exactly the same place on every trial of the block. However, the multiplex was rearranged at the start of each new block. In the conditions in which scenes were not arranged by category, scene placement in the multiplex was randomly determined at the start of the block. In conditions in which scenes were arranged by category, each category of scenes was grouped to a $3\times 3$ portion of the array (corresponding to the left, centre or right sections of the multiplex), with the allocation of categories to portions of the multiplex being fully counterbalanced between participants. Within categories, allocation of scenes to positions in the $3\times 3$ section of multiplex was randomly determined at the start of the block. At the start of each block participants were informed that they were beginning a new block, but were given no information about the experimental manipulations. They were informed that the locations of scenes within the multiplex would differ from the previous block but would be the same on every trial of the block.

#### Data analysis

Participants who performed poorly (< 70% correct over the entire experiment) were excluded from subsequent analyses (N removed = 1). For the remaining 35 participants the mean proportion of trials on which the target letter was reported correctly was 0.91 (SD = 0.28). Only response times for correct trials are included in the analyses that follow. Response times were log-transformed and outliers more than 2.5 standard deviations from the log-transformed mean response time were excluded (84 trials removed, 1.46% of correct trials).

Data were analysed using linear mixed models (LMMs) using the lme4 package (Bates et al. 2015) in the R statistical programming environment (R Core Team 2020). Fixed effects of border presence and scene organisation within the array were sum coded in order to allow interpretation of main effects and interactions in a similar manner to traditional ANOVA approaches while maintaining the advantages of LMMs in terms of estimating by-item and by-participant variance simultaneously. The package lmerTest (Kuznetsova et al. 2017) was used to calculate *p* values, and ggplot2 (Wickham et al. 2016) was used for plotting data.

**Fig. 4:**
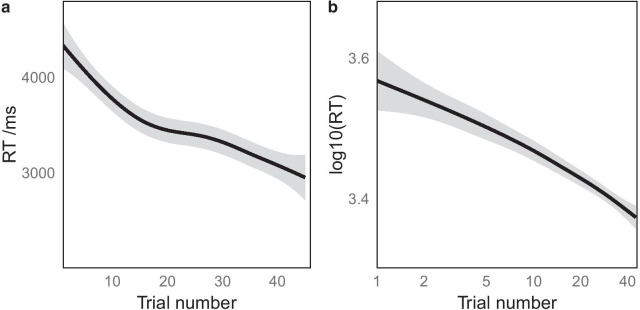
Response time and trial number. **a** raw response time in ms plotted against ordinal trial number within each block. The plot shows a nonlinear relationship with RT decreasing more rapidly over the first few trials than later in the block. **b** The same data plotted with log-transformed RT on the y-axis and with the x-axis plotted in a logarithmic scale. In this plot the relationship is close to linear

At the start of each block, positions of individual scenes within the array are unknown by the participant, but because the arrangement of scenes in the multiplex array is repeated across all trials in a block, participants are highly likely to get faster over the course of the block. Therefore trial number was included as a fixed effect in the models in order to remove any effects of this from the analysis of the two experimental manipulations. Visual inspection of the effect of trial number on RT within blocks showed a nonlinear effect (Fig. 4a), but log-transforming the trial number (and RT) resulted in an approximately linear trend (Fig. 4b). Therefore log(trial number) was used as the fixed effect in the LMMs reported below.

With a lack of a priori power calculation for the LMM analysis conducted, post hoc power was estimated using the R package simr (Green and MacLeod 2016) to run power simulations from the LMM as suggested by Kumle et al. (2020) and Brysbaert and Stevens (2018). The PowerSim() function was used to calculate power for each fixed effect in the LMM across 1000 simulations. The effect of having fewer or more participants on power was simulated for each fixed effect in the model by using the extend() and powerCurve() functions in simr. These power curves allow an estimate of the minimum sample size to detect effects of each fixed effect in the model and can be used for a priori sample size and power calculations in later experiments.

### Results

The LMM reported in Table 1 included the interaction between multiplex organisation and border presence, but did not include interactions between these factors and log-transformed ordinal trial number. This is because adding in these interactions did not significantly improve the fit of the model, $\chi^{2} = 1.3, {df} = 3, {p} = 0.729$.

**Fig. 5:**
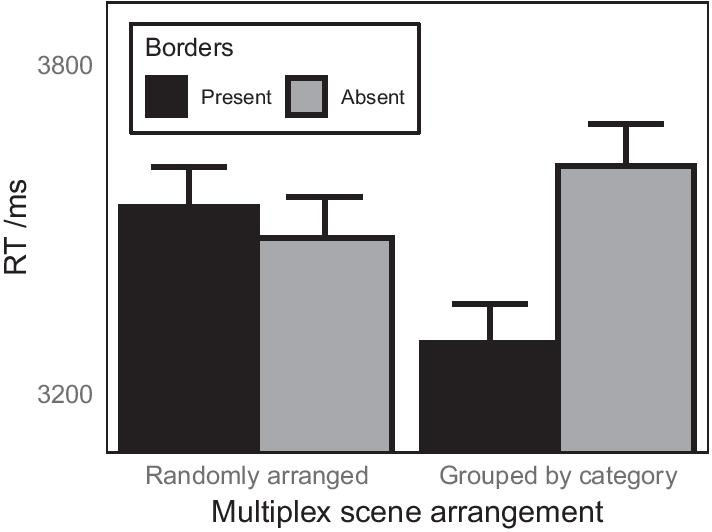
Effects of multiplex organisation and border presence between scenes on response time when searching for a target scene in the multiplex for Experiment 1. The plots show means derived from the raw data and therefore do not partial out effects due to other variables in the linear mixed model. Error bars show 1 standard error of the mean

There was no overall effect of whether scenes were arranged by semantic category, but there was a significant overall effect of whether or not borders were present between scenes in the multiplex, with faster responses when borders were present between scenes ($M = 3416$ ms, SD = 2729 ms) than when scenes were not separated by borders ($M = 3550$ ms, SD = 2863 ms). This effect was qualified by a significant interaction with scene arrangement (Fig. 5).

The power of the LMM to find effects for each of the fixed effects of interest was estimated by running simulations from the observed data and by simulating power across different numbers of participants. The estimates of power, derived from 1000 simulations of the LMM, were 89.5% (95% CI 87.4–91.3%) for the fixed effect of arrangement, 93.9% (95% CI 92.2–95.3%) for the fixed effect of border presence and 90% (95% CI 88–91.8%) for the interaction between these two factors. Power curves for simulated power across different numbers of participants are shown in “Appendix”.

**Table 1:** Results of LMM to predict log-transformed response time in Experiment 1

Effect	*b*	SE	*t*	*df*	*p*
Intercept	3.59	0.024	150.49	96.01	< .001
Arrangement	− 0.01	0.004	− 1.42	5594.88	.155
Border presence	− 0.01	0.004	− 2.09	5594.08	.037
log10(trial no.)	− 0.12	0.010	− 12.70	5596.46	< .001
Arrangement * Border presence	− 0.01	0.004	− 3.20	5594.11	.001

A follow-up LMM was run to explore the significant interaction between scene arrangement and the presence of borders with contrast coding to test the simple effects of border presence within each type of organisation. When the scenes were randomly positioned within the multiplex, having borders between scenes made no difference to the response time, ${b} = -0.01$, SE = 0.010, ${t} = -0.79, {p} = .430$. When scenes were arranged by category, participants were faster to find the target scene when scenes were separated by borders than when they were touching, ${b} = 0.04$, SE = 0.010, ${t} = 3.75, {p} < .001$. Looking at simple effects of organisation in the multiplex, an LMM showed that in the absence of borders between scenes, there was no benefit for arranging scenes by semantic category in the multiplex, ${b} = -0.01$, SE = 0.010, ${t} = -1.26, {p} = .208$. However, when there were gaps present between scenes, participants were faster to find the target when scenes were grouped by category than when they were randomly arranged, ${b} = 0.03$, SE = 0.010, ${t} = 3.27, {p} = .001$.

### Discussion

The results of Experiment 1 provide a first empirical demonstration that grouping scenes may benefit search in a CCTV multiplex, providing support for previous recommendations that camera feeds in the multiplex should be arranged logically (Donald 1998; Pikaar et al. 2015; Wallace and Diffley 1998) and reports by CCTV operators that they find it difficult to find scenes when faced with ad hoc multiplex layouts in the CCTV control room (Keval and Sasse 2010). Previous studies have failed to find a benefit by arranging scenes logically within a multiplex (Harris et al. 2008; Stedmon et al. 2011), but have organised only a limited number of scenes and done so by geographical proximity. In the present study, scenes were grouped by semantic category rather than geographical proximity and the multiplex array was considerably larger than in previous evaluations of the effects of logical layouts. It may be that arranging by semantic category is a better organising principle than geographic proximity as the semantic categories of multiple scenes can be processed rapidly in peripheral vision (Potter and Fox 2009; Rousselet et al. 2004; VanRullen et al. 2004) and thus this peripherally available information can be used to guide search to specific portions of the multiplex array.

In order for participants to selectively benefit from grouping scenes by semantic category in the multiplex, participants must have been learning more about the multiplex than simply where each scene was located. The arrangement within the multiplex was constant across trials within each block, providing the opportunity for participants to benefit from mere repetition of targets that occur at repeated locations, within a repeated context. Such search benefit from repeated target-context associations is well-known in the visual search literature for arrays of simple targets (Chun and Jiang 1998, 2003) and arrays of more realistic objects (Hout and Goldinger 2010). Certainly in the present study participants benefited from repeatedly searching the same array, with response times reducing over trials throughout each block. But the finding that search times were also shorter when the scenes in the multiplex were arranged by semantic category demonstrates that the search benefit in these situations was more than that from simply repeating the same multiplex on each trial. Rather, participants appear to have been able to utilise the groupings within the multiplex to speed search. Participants can learn to ignore irrelevant parts of a search array when targets only appear within certain parts of the array (Kunar et al. 2008) and detecting changes in multiplexes is faster if participants are able to ignore irrelevant scenes within the multiplex (Stainer et al. 2017). However, in Experiment 1 it was not simply the case that portions of the display could be ignored because different parts of the multiplex were relevant depending on the identity of the target. The findings from Experiment 1, therefore, are more similar to what has been found within single scenes: that participants search the regions of a scene where they expect to find the target, and search different regions depending upon the target they are searching for (Ehinger et al. 2009; Torralba et al. 2006).

When interpreting the effect of grouping found in this experiment, it should be noted that while scenes were selected and grouped based on their semantic category, such grouping may also lead to other differences between the groups of scenes including differences in basic visual characteristics, and these differences may contribute to the search benefits found in Experiment 1. This co-variation of basic visual properties and scene semantics is likely to be the case for natural scenes, given the correlations between second-order scene statistics and scene categories (Torralba and Oliva 2003), and the likely role of scene statistics in discerning the gist of a scene (Oliva and Torralba 2006). The extent to which search benefits arise from semantic understanding or other differences that correlate with these scene groupings cannot be determined in the present study because visual characteristics were not controlled in selecting the stimuli. However, the categories of scenes used in the present study were all drawn from the same superordinate category of urban scenes. The specific categories do not correspond to well-differentiated basic categories of scenes, but rather reflect meaningful ways to group scenes given the set of surveillance images available. The selected groupings (city centre, suburban and traffic) also reflect types of scene that are differentiated in terms of the likely types and times of crime (e.g. Ratcliffe 2010) and therefore represent sets of scenes that are likely to be associated different monitoring priorities in real-world CCTV surveillance. Because the categories are all drawn from the same superordinate category, they are unlikely to differ greatly in terms of their visual properties and all lie in a similar space along the openness and naturalness axes proposed by Torralba and Oliva (2003). For this reason, basic visual properties are unlikely to be the main contributing factor underlying any effects of arranging scenes by category, and effects are therefore interpreted as reflecting semantic processing of scenes.

The findings from Experiment 1 demonstrate that participants can spontaneously (without any explicit information about organisation within the multiplex) utilise the association between the category of a briefly presented target scene, and clusters of scenes within the multiplex that all share the same category as the target. It is important to note, however, that this benefit for searching a multiplex of scenes that are organised by category was only found when the individual scenes were separated by borders; when the scenes touched each other, whether scenes were arranged randomly or by category made no difference to how long it took to find a specific scene. It may be that the gaps between scenes helped segment individual scenes to assist for semantic processing in peripheral vision, in the same way that objects are easier to recognise in peripheral vision when isolated (Davenport and Potter 2004), or on a noise background (Leroy et al. 2020), than when embedded in a scene. In this way, each individual scene is processed more easily in peripheral vision and this helps participants to use this peripheral processing of scenes better to aid search. However, the lack of effect of borders when scenes were randomly arranged argues against this interpretation that the effect of borders is simply a result of better (semantic) processing of individual scenes in peripheral vision. If this were the case, there should have been a similar advantage for processing each scene when separated by borders irrespective of the arrangement of scenes in the multiplex, but there was not. Rather, it would seem that the benefit offered by having borders between scenes is to help participants to utilise the potential efficiencies offered when searching a multiplex in which scenes are organised by category. Perhaps, visually segmenting the multiplex into individual scenes allows sufficient peripheral processing of individual scenes to enable the viewer to identify that regions of the multiplex share common semantics and thus guide search to this region of the multiplex and therefore reduce search times. When scenes are not separated by borders, the lack of visual distinction between scenes might hinder peripheral processing of individual scenes to the extent that regions of common semantics between scenes are not identified.

The result that benefits of arranging the scenes by category were only found when the scenes were also separated by borders between them has practical implications in the CCTV control room. In modern systems, in which scenes can be rearranged and organised in particular ways, scenes often abut and this in itself may reduce or remove any benefit that the operator may get from organising the scenes in particular ways. Thus, it seems that (re-)introducing physical separation between each scene in the multiplex would be a useful recommendation for systems in which scenes are arranged logically.

While the results of Experiment 1 provide empirical grounds for recommending the above configuration within a CCTV multiplex, it is important to note that participants in Experiment 1 were not informed that in some blocks scenes would be arranged by category. This was in order to see whether arranging the scenes in the multiplex offered any spontaneous benefit for search, an important first step in understanding the role of arrangement on search in the CCTV multiplex. However, in CCTV control rooms, organisation principles are not hidden from the operators and are made explicit to them (indeed the teams of operators themselves may choose their own arrangement of scenes for their shifts in some control centres). Experiment 2 was conducted in order to test the potential benefit of organising the multiplex by semantic category—and whether there is a need to separate the individual scenes with borders—under the more realistic situation of participants knowing in advance how the multiplex is arranged.

## Experiment 2

Knowing in advance—either from experience or from simply being told—that particular sets of scenes are located in specific parts of the CCTV multiplex is very likely to aid search for any particular scene, provided that the operator can easily make the association between the searched-for scene and the logical groups within the multiplex. Indeed, when participants expect an object to be in a particular region of a scene, they will preferentially search within that region (Ehinger et al. 2009; Torralba et al. 2006), even from the first eye movement after the scene appears (Spotorno et al. 2014). These expectations can come from prior experience—as in the studies cited above—or can be novel, based solely on information given to the participants during the experiment (Zelinsky and Schmidt 2009).

Experiment 2 followed the same design as Experiment 1, with the exception that participants were explicitly informed at the start of blocks whether scenes would be arranged by category and, if so, were informed where in the array each category of scenes would be located. This second experiment offers a test of how arranging by category benefits search when the arrangement is made explicit to the observer and whether the presence of borders is again necessary to gain this benefit.

### Methods

#### Design

As in Experiment 1, with the exception that the manipulation of scene arrangement in Experiment 2 was revealed to participants (see procedure below), whereas it had remained hidden to participants in Experiment 1.

#### Participants

Thirty-six participants took part in the experiment. None of the participants had taken part in Experiment 1. All participants were undergraduate students and took part voluntarily or for course credit. Participants were recruited by word of mouth and through the SONA recruitment system. All participants had normal or corrected-to-normal vision. Participants were naive to the purposes of the study. The study was approved by the local Psychology ethics committee (PEC/3510/2016/9).

Using the effect sizes expected from Experiment 1, simulations of power using the data from Experiment 1 were run using the simr package in R to estimate the power across different number of participants (see “Appendix”). These simulations suggested a minimum sample size of 28 would be required to achieve at least 80% power for all fixed effects in Experiment 2. The recruited sample size of 36 ensures higher power to detect all effects of interest even after any necessary exclusions of participants during analyses.

#### Materials

As in Experiment 1 with the following exceptions. Because the categories were to be revealed to participants, having easily labelled categories with clear membership for each scene was more important than it had been in Experiment 1. In discussion with a group of six third-year undergraduate psychology students who assisted with data collection for this study, it was decided that several of the scenes used in Experiment 1 were somewhat difficult to assign to the categories of scenes in which they were placed, especially if viewed for only a short time. The following problems were identified and rectified by selecting alternative images from the 85 originally supplied by Police Scotland.

*City centre scenes* Two of the city centre images contained empty streets that could be confused for the quiet suburban areas and one included a busy road with traffic, that could be confused for a busy traffic scene. These three scenes were replaced with scenes that clearly showed shop fronts and did not include busy roads. The category was re-labelled as *Shopping areas* to clarify the content as all 9 members contained clearly visible shop fronts.

*Suburban scenes* This category proved the most difficult because it included a mix of scenes, comprising quiet roads with little else visible (3), residential streets (3), parking areas (2) and commercial buildings (1). The parking areas and commercial building might be confusable with city centres. Of the scenes that included views of roads, three included more than five parked cars at the roadside, which might be confused with the traffic scenes category. Given the heterogeneity of this category and its possible overlap with the other categories, a new category was formed or Experiment 2 that was more homogeneous and easily distinguishable from the other categories of scenes. Three of the original images from this set plus another six images were combined to create a category of *Quiet roads* in which fewer than 4 cars were visible (whether parked or moving).

*Traffic scenes* Five of the scenes selected for this category contained fewer than five cars and so it was felt that these might be confused with quiet suburban roads that were present in some of the suburban image category used in Experiment 1. Five replacement scenes were used to ensure that all scenes contained foregrounded views of roads with more than five cars visible. For the participants, this category was labelled as *Busy roads*.

#### Procedure

**Fig. 6:**
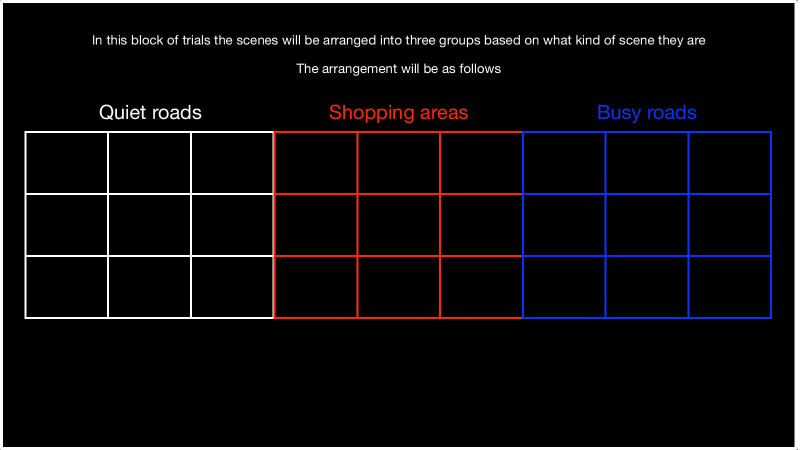
Instructions shown to participants at the start of blocks when scenes were arranged by category. As in Experiment 1, assignment of categories to regions of the multiplex was fully counterbalanced across participants, so this example shows one of 6 possible orderings of the three scene categories in the multiplex

As in Experiment 1, with the following exceptions. The search target scene was displayed for 1000 ms in Experiment 2, rather than 750 ms as in Experiment 1. At the start of blocks in which the scenes were grouped by category, participants were shown a screen that explained this and displayed where each of the three categories of scenes would be in multiplex. The example shown in Fig. 6 is for the condition in which there were no borders between images. In blocks where borders were present, the spacing between the outlines in the instruction screen was adjusted to reflect this. In this way, the locations outlined in the instruction screen precisely corresponded with the locations of the scenes in the multiplex shown on each trial of the block that followed.

#### Data analysis

Participants who performed poorly (< 70% correct over the entire experiment) were excluded from subsequent analyses (N removed = 1). For the remaining 35 participants the mean proportion of trials on which the target letter was reported correctly was 0.92 (SD = 0.26). Only response times for correct trials are included in the analyses that follow. Response times were log-transformed and outliers more than 2.5 standard deviations from the log-transformed mean response time were excluded (74 trials removed, 1.27% of correct trials).

LMMs were used to analyse response times and were constructed in the same manner as Experiment 1, with fixed effects of arrangement, border presence and trial number, coded in the same way as in Experiment 1.

### Results

**Fig. 7:**
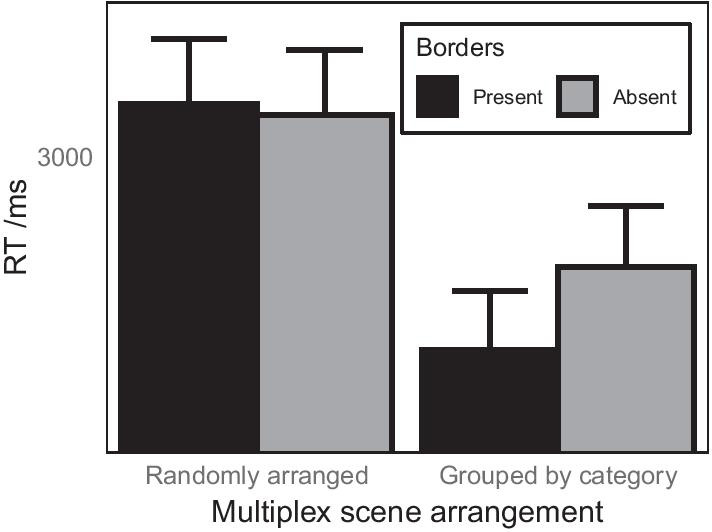
Effects of multiplex organisation and border presence between scenes on response time when searching for a target scene in the multiplex for Experiment 2. The plots show means derived from the raw data and therefore do not partial out effects due to other variables in the linear mixed model. Error bars show 1 standard error of the mean

The pattern in the data plotted in Fig. 7 was similar to that found for Experiment 1. For multiplexes with randomly arranged scenes, whether or not there were borders between scenes made little difference to response times. For multiplexes in which scenes were grouped by category, response times were numerically faster when individual scenes were separated by borders. However, it is also evident from this plot that there was an overall effect of scene arrangement that was not found in Experiment 1, with response times faster when scenes within multiplexes were arranged by category. The output for an LMM to predict response time from the arrangement of scenes and presence of borders in the multiplex array is shown in Table 2. As in Experiment 1, adding in the interactions between log-transformed trial number and the other fixed effects did not improve the fit of the model, $\chi^{2} = 2.04, {df} = 3, {p} = 0.564$, and so the model reported here does not include these interactions.

The LMM confirmed the overall effect of arrangement described above, with response times being significantly faster when scenes were arranged by category ($M = 2853$ ms, SD = 2241 ms) than when scenes were randomly positioned within the multiplex ($M = 3047$ ms, SD = 2371 ms). However, there was no main effect of the presence of borders in the multiplex, nor was there a significant interaction between these factors (Fig. 7).

**Table 2:** Results of LMM to predict log-transformed response time in Experiment 2

Effect	*b*	SE	*t*	*df*	*p*
Intercept	3.51	0.023	150.68	80.85	< .001
Arrangement	− 0.01	0.003	− 2.90	5688.50	.004
Border presence	0.00	0.003	− 1.03	5688.10	.303
log10(trial no.)	− 0.11	0.009	− 12.13	5689.07	< .001
Arrangement * Border presence	0.00	0.003	− 1.07	5689.88	.283

### Discussion

Experiment 2 showed that when participants were explicitly informed that the scenes in the multiplex were arranged by category and where each category would appear in the multiplex, participants gained a clear advantage from this when searching for a target scene. From a theoretical point of view this result is unsurprising given what is known about how observers typically restrict search to regions of a scene in which they expect the target to appear (Ehinger et al. 2009; Kanan et al. 2009; Torralba et al. 2006), that this can happen even when these expectations are set up on-the-fly by information given in the experiment rather than from any *a priori* knowledge (Zelinsky and Schmidt 2009), and that these regions can be identified in peripheral vision and associated with the search target within the timescale of planning the first saccade after a scene appears (Spotorno et al. 2014). What the current study demonstrates for the first time in this respect is that observers’ ability to utilise semantic analysis of regions within a scene to aid search generalises to multi-scene viewing, where the “region” comprises a set of scenes drawn from a common semantic category.

In Experiment 1, the presence of borders between scenes resulted in faster response times for multiplexes of scenes that were grouped by category but made no difference for randomly arranged multiplexes. In Experiment 2, despite a numerically similar pattern to that found in Experiment 1, having borders between scenes did not offer any search benefit. It seems therefore that separating scenes by borders may not be necessary when the arrangement of scenes in the multiplex is known to the observer, as is usually the case in the CCTV control room.

In both Experiments 1 and 2, the frequency of targets was the same across the three categories of scenes. This means that over the course of each block, all locations within the multiplex were equally likely to be searched. While this design is useful for establishing the benefits of grouping scenes by semantic category, it fails to capture a key aspect of search within the CCTV control room: that some scenes will more often be the target of reactive or proactive surveillance. This inequality between scenes in the multiplex is due to the fact that crime likelihood and frequency vary over space and time (Chainey and Ratcliffe 2013; Ratcliffe 2010). Experiment 3, therefore, includes a manipulation of how frequently each category of scenes is drawn upon to provide a search target for each trial, such that one category more commonly provides the search target than the others. This design allows the search benefits (and potential costs) of organising scenes by semantic category in the multiplex in the more realistic situation of searching frequently for some scenes and rarely for others.

## Experiment 3

The fact that different types of crime will be more likely at different times of day, and that these crimes each have their own non-uniform distribution over space (Felson and Poulsen 2003; Gupta et al. 2012; Ratcliffe 2010) means that the most frequently monitored scenes in the CCTV control room will vary over the 24-h surveillance cycle (Stainer et al. 2013). The search advantages for finding target scenes in multiplexes grouped by scene category are likely to offer particular benefits for the most frequently monitored categories of scenes: grouping together the scenes that operators most often need to find and monitor is likely to be beneficial for both proactive and reactive surveillance tasks.

The unequal frequency of monitoring different categories of scenes raises an important question about the possibility of grouping scenes in the multiplex by semantic category: while this principle can offer obvious benefit for supporting search amongst the most frequently monitored types of scenes in the multiplex array, does it result in a cost for monitoring the groups of scenes where crime is unlikely?

When human observers are required to search for targets that are rarely present in the scenes that they are searching, detection performance can be very poor (Wolfe 2020; Wolfe et al. 2005), and response times are longer (Laberge and Tweedy 1964), an effect amplified when available attentional resources are limited (Hon and Tan 2013). This alone might impact an operator’s ability to detect events in locations where crime rarely occurs or to orient to these camera feeds when required to, irrespective of whether scenes are arranged logically or randomly. However, this difficulty for such low-prevalence scenes may be exacerbated by grouping scenes by semantic category in the multiplex. This is because getting close to a target during search can be sufficient to allow extra-foveal detection of the target: search can be modelled as a random walk in which the target is detected once the random walk brings the eye close enough to it (Clarke et al. 2016; Nowakowska et al. 2017). In randomly arranged multiplexes, the chances of looking at a screen close to one of the rarely monitored scenes is likely to be higher than when the multiplex is grouped by scene type. In the case of the former, rarely monitored camera feeds may be close to more frequently monitored scenes, whereas by grouping scenes of the same type together, it is more likely that these rarely monitored scenes will be next to other rarely monitored scenes, creating a region of the CCTV multiplex that is looked at much less than other regions of a scene (Kanan et al. 2009; Torralba et al. 2006). The result of this could be lower detection of crime in such locations during proactive surveillance and slower response times to calls for information about such locations in reactive surveillance. Both of these may be costly for effective CCTV surveillance. Indeed, Hodgetts et al. (2018) found that when participants spontaneously adopted a strategy of monitoring some scenes more than others, participants missed more events than when they monitored each scene equally. Experiment 3 provides an empirical test of the costs and benefits to search times for high- and low-prevalence target scenes in randomly arranged multiplexes and multiplexes with scenes grouped by category.

### Methods

#### Design

The experiment followed a $2\times 2$ repeated-measures design with two independent variables: the organisation of scenes in the multiplex (mixed randomly or organised by semantic category) and the distribution of target frequency across the three categories of scenes (equal or unequal). As in previous experiments, the dependent variable was manual response time.

#### Participants

Thirty-six participants took part in the experiment. None of the participants had taken part in Experiments 1 or 2. All participants were undergraduate students and took part voluntarily or for course credit. Participants were recruited by word of mouth and through the SONA recruitment system. All participants had normal or corrected-to-normal vision. Participants were naive to the purposes of the study. The study was approved by the local psychology ethics committee (PEC/3510/2016/9).

There were no prior data from which to simulate power for detecting effects of varying target frequency in Experiment 3. Therefore, an artificial dataset was created which replicated the design of Experiment 3; this artificial dataset was used to simulate models and power. For the model, the grand mean and fixed effects of arrangement and trial number were taken from Experiment 2. For fixed effects of target frequency and interactions involving this factor, fixed effects were estimated based on the assumptions that (1) effects of varying target frequency were likely to be stronger than those arising from scene arrangement and (2) that high-frequency targets would be responded to quicker and low-/very low- frequency targets would be responded to slower than the conditions in which all targets were equally likely (see “Appendix” for more details). Assuming small but present interactions between target frequency and scene arrangement resulted in a simulated power of 90% (95% CI 88–91.8%) for 36 participants (requiring a minimum of 27 participants for 80% power). Power for detecting effects of target frequency increased for 36 participants under assumptions of increased effects of target frequency, larger interactions or no interactions (see “Appendix” for details).

#### Materials

Materials were exactly the same as in Experiment 2.

#### Procedure

As in Experiment 2, but with the following exceptions, each of the four blocks in the experiment comprised 60 trials. In blocks in which the target frequency was equally distributed across scene categories, there were 20 trials in which the target was selected from each of the three categories of scenes (it was a randomly selected member of the category on each trial). In blocks in which the target frequency was unequally distributed across scene categories, one category was selected to be the target category on 42 trials (70% of trials), a second category was selected for 12 trials (20%), and a third category was the target for 6 trials (10%). Allocation of categories to each of the three target prevalences in these blocks was counterbalanced across participants using a Latin square. Participants were informed at the start of each block whether target scenes were equally likely to be from any category (“In this block the target is *equally likely* to appear in any type of scene”) or was more likely to be drawn from one particular category (e.g. “In this block the target will *usually* appear in one of the *shopping area scenes*”). The specific frequencies of target prevalence across categories were not revealed to participants. The assignment of categories to target prevalence conditions remained fixed across blocks within each participant—such that if shopping centres were assigned as the high-target-prevalence category, they remained with this assignment in both of the blocks that had unequal target frequencies across scene categories.

#### Data analysis

Participants who performed poorly (< 70% correct over the entire experiment) were excluded from subsequent analyses (N removed = 1). For the remaining 35 participants the mean proportion of trials on which the target letter was reported correctly was 0.92 (SD = 0.28). Only response times for correct trials are included in the analyses that follow. Response times were log-transformed, and outliers more than 2.5 standard deviations from the log-transformed mean response time were excluded (119 trials removed, 1.55% of correct trials).

An LMM was used to analyse response times and was constructed in a similar manner to those in Experiments 1 and 2. As before, the model included fixed effects of arrangement (randomly arranged, grouped by category), which was sum coded, and trial number, which was log-transformed. The LMM also included the fixed effect of target frequency (baseline, high, low and very low), which was coded for simple effects to compare each of the high-, low- and very low-target frequency categories to the baseline condition in which the target was equally frequently drawn from each scene category.

### Results

**Table 3:** Results of LMM to predict log-transformed response time in Experiment 3

Effect	*b*	SE	*t*	*df*	*p*
Intercept	3.57	0.022	162.85	101.07	< .001
Arrangement	− 0.01	0.005	− 1.91	7510.45	.056
Baseline versus high frequency	− 0.06	0.007	− 8.98	7513.92	< .001
Baseline versus low frequency	0.03	0.011	3.19	7512.41	.001
Baseline versus very low frequency	0.06	0.014	4.28	7511.95	< .001
log10(trial no.)	− 0.15	0.008	− 18.39	7510.89	< .001
Arrangement * Baseline versus high frequency	0.01	0.007	1.05	7513.29	.292
Arrangement * Baseline versus low frequency	0.02	0.011	1.85	7512.13	.064
Arrangement * Baseline versus very low frequency	0.03	0.014	2.27	7511.67	.023

**Fig. 8:**
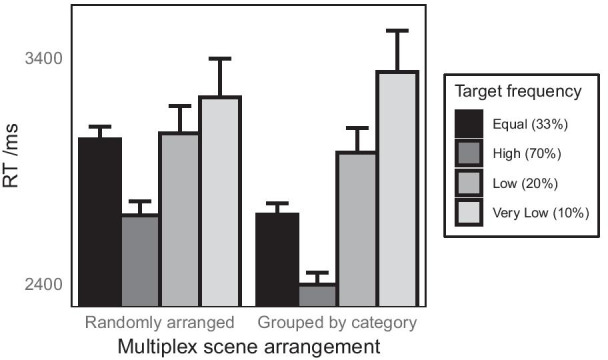
Effects of multiplex organisation and target (category) frequency on response time when searching for a target scene in the multiplex for Experiment 3. The plot shows means derived from the raw data and therefore do not partial out effects due to other variables in the linear mixed model. Error bars show 1 standard error of the mean

A model comparison showed a significant difference between an LMM that included the interactions between log-transformed trial number and the other fixed effects in the model, $\chi^{2} = 14.93$, ${df} = 7, {p} = 0.037$. However, comparing BIC across the two models showed that the model which included all interactions was a significantly poorer fit to the data (BIC = 1770.02) than the model which did not include interactions with trial number (BIC = 1722.42). Therefore the model without interactions is reported here.

The LMM (Table 3) showed that overall, compared to the baseline condition, in which all target categories were equally likely ($M = 2871$ ms, SD = 2274 ms), response times were significantly faster for high frequency targets ($M = 2549$ ms, SD = 2135 ms) and significantly slower for both low ($M = 3025$ ms, SD = 2260 ms) and very low ($M = 3282$ ms, SD = 2443 ms) frequency target scenes. These overall effects of target frequency were qualified by an interaction with multiplex arrangement for very low-frequency targets (compared to the baseline) and an approaching significance interaction for low-frequency targets, indicating that the response times for these rarer target scenes were effected by multiplex structure (Fig. 8).

**Table 4:** Results of LMM to predict log-transformed response time in Experiment 3, separated by multiplex arrangement

Effect	*b*	SE	*t*	*df*	*p*
Randomly arranged multiplexes					
Intercept	3.58	0.025	142.65	165.48	< .001
Baseline versus high frequency	− 0.06	0.010	− 6.35	3699.86	< .001
Baseline versus low frequency	0.02	0.016	1.10	3692.83	.270
Baseline versus very low frequency	0.03	0.021	1.49	3689.89	.136
log10(trial no.)	− 0.15	0.012	− 12.56	3684.73	< .001
Categorically grouped multiplexes					
Intercept	3.56	0.026	138.32	131.24	< .001
Baseline versus high frequency	− 0.05	0.009	− 5.89	3771.36	< .001
Baseline versus low frequency	0.06	0.014	4.00	3770.83	< .001
Baseline versus very low frequency	0.10	0.019	5.07	3770.95	< .001
log10(trial no.)	− 0.15	0.011	− 13.68	3771.31	< .001

Follow-up LMMs were run evaluate the effect of target frequency separately for randomly arranged multiplexes and multiplexes arranged by scene category (Table 4). For randomly arranged multiplexes, response times were faster for high-frequency targets than the baseline, but low- and very low-frequency targets did not differ from the baseline condition in which all targets were equally likely. For multiplexes with scenes arranged by category, response times were faster for high-frequency targets and slower for both low- and very low-frequency targets compared to the baseline condition.

A further LMM coded for simple effects of multiplex arrangement within each frequency of target showed that compared to randomly arranged multiplexes, multiplexes with scenes arranged by category resulted in faster response times in the baseline condition in which all scene categories were equally often the target, ${b} = 0.05$, SE = 0.009, ${t} = 5.37, p< .001$, and for high-frequency targets, ${b} = 0.03$, SE = 0.010, ${t} = 3.16$, ${p} = .002$. No significant effect of multiplex arrangement was found for low-frequency targets, ${b} = 0.01$, SE = 0.019, ${t} = 0.40$, ${p} = .689$, or very low-frequency targets, ${b} = -0.02$, SE = 0.027, ${t} = -0.67, {p} = .501$.

### Discussion

The results from Experiment 3 show that, as expected, people are faster to search for target scenes, when the target is drawn frequently from the same category. The fact that this was found both for randomly arranged multiplexes and multiplexes with scenes grouped by category is not surprising and is consistent with previous findings for search within single scenes that has shown that repeating a target at the same position in a scene results in increasingly faster searches (Brockmole et al. 2006; Brockmole and Henderson 2006). This result of course also suggests that in any type of arrangement of a multiplex for CCTV surveillance, finding a particular scene in the array will be fastest for the scenes that are most often monitored (that is, where crime is most likely to occur). Furthermore, there were overall costs for searching for the rarer targets: low- and very low-frequency targets took longer to find compared to the same scenes in the baseline condition. Such a cost for finding rare targets is consistent with previous literature that has suggested that rare targets are more frequently missed (Wolfe 2020; Wolfe et al. 2005), and take longer to correctly detect (Hon and Tan 2013; Laberge and Tweedy 1964) compared to common targets. Unlike the previous two experiments there was not an overall effect of arranging scenes by category in the multiplex (although it was close to significant). Rather in the overall model, the effect of scene arrangement only contributed significantly through an interaction with target frequency. This indicates that any benefit or detriment of arranging scenes may be found selectively within certain groups of scenes, depending on how frequently they are searched. The follow-up analyses that consider this interaction in more detail are therefore the key analyses for understanding how arranging scenes in a multiplex by category might influence search times when certain categories are more likely to be searched than others.

A particular concern that motivated Experiment 3 was whether arranging the scenes in the multiplex by category might result in a significant cost for finding the rarest target scenes; if so this cost might outweigh the potential benefits to search for organising the multiplex in this way. The finding that there were costs to search times for targets drawn from the low- and very low-frequency scene categories when multiplexes were arranged by scene semantics, but no such costs for these rare targets in randomly arranged multiplexes might appear to support this concern. However, comparison to the baseline condition of having all scene categories equally likely to be the target may not be the correct comparison for most real-world surveillance scenarios. This is because in most surveillance tasks there will be an unequal probability that each scene will require proactive monitoring or reactive orienting to, due to the variability in crime across space and time (Ratcliffe 2010); thus, it will rarely be the case that at any time or for any task, the probability of needing to attend to all types of scene will be equal. A more appropriate comparison is to consider the differences between randomly arranged and categorically grouped multiplexes when the target prevalence varies between the categories of scene. Search times were faster for the most prevalent target category when the multiplex was arranged by scene category than when it was randomly arranged. However, for the categories that had low and very low target frequency, there were no significant differences in search times between the two types of multiplex arrangement.

The results of Experiment 3 appear at odds with prior work by Hodgetts et al. (2018) which showed that participants who prioritised some scenes over others during a CCTV surveillance task had a lower detection rate for incidents than those who monitored all scenes equally. The apparent difference may arise because the present study and this prior work focused on different aspects of the surveillance task; the present study focused on the time to search for a specific scene, whereas Hodgetts et al. (2018) measured the probability of detecting an incident. Thus, the costs of prioritisation found in Hodgetts et al. (2018) may reflect costs for processes that occur after selection of a scene, which are not detectable in the present study due to the nature of the paradigm. However, this need not undermine the potential benefit offered by arranging scenes by category when operators need to orient rapidly to a specific scene in the multiplex.

The findings from Experiment 3, therefore, suggest that compared to a randomly arranged multiplex, one that has scenes grouped by category provides search benefits for the most frequently searched-for category of scenes, but no significant search costs for the more rarely search-for scene categories. While this provides further evidence in support of arranging scenes within multiplexes by their category, it should be noted that statistical confidence in the estimates of search times for low- and very low-frequency targets is necessarily lower than that for high-frequency targets. The increased variability in these conditions is evident in the plot of the data and makes any assessment of the differences between the two types of multiplex arrangement for these categories of scenes harder to identify statistically. The reported finding of no cost for rare targets in categorically arranged multiplexes compared to randomly arranged multiplexes must therefore be treated with caution.

## General discussion

The present study employed a novel scene-search paradigm that most closely models how operators search for a specific scene in the multiplex during reactive surveillance but is also relevant to aspects of proactive surveillance. The paradigm has the advantage of using more extensive multiplexes—with 27 scenes—than previous laboratory-based paradigms for empirical investigations of multiplex surveillance. Furthermore, scenes were static frame grabs from CCTV cameras in one Scottish city, supplied by Police Scotland, ensuring that images in the multiplex accurately reflected the scale and viewpoint of real camera feeds in police CCTV control rooms. This paradigm was used to evaluate the possible search benefits of arranging scenes in the multiplex into groups according to their semantic categories.

Across three experiments, consistent evidence was found that searching for a scene within the multiplex was faster when the scenes in the multiplex were grouped by semantic category. Previous studies have found no benefit for arranging scenes by geographical logic (Harris et al. 2008; Stedmon et al. 2011) despite recommendations that organising the multiplex logically should help operators (Donald 1998; International Organization for Standardization 2013; Pikaar et al. 2015; Wallace and Diffley 1998) and reports from CCTV operators that randomly arranged multiplexes are hard to search through Keval and Sasse (2010).

The benefit for organising scenes within the multiplex found in the present study may have arisen from at least three potential causes. First, it may be that previous paradigms were not challenging enough, having too few scenes in the multiplex for logical organisation to offer sufficient benefit (Harris et al. 2008; Stedmon et al. 2011). Second, it may be due to differences in the nature of the operator’s task, which was to locate and report a detail from a target scene in the present study, whereas previous studies have typically used tasks of following a suspect (Harris et al. 2008; Stedmon et al. 2011) or monitoring for suspicious behaviour (Howard et al. 2013). Third, it may be that grouping scenes by semantic category is a more beneficial method of organising the multiplex than grouping scenes by geographical proximity.

From a theoretical perspective, it is reasonable to suggest that grouping scenes by semantic category should support multiplex search. A scene’s semantic category can be extracted very rapidly (Biederman 1972; Potter 1975, 1976) and can be extracted for several scenes when they are presented simultaneously in peripheral vision (Potter and Fox 2009; Rousselet et al. 2004; VanRullen et al. 2004). Therefore, semantic information about scenes in the multiplex is available in peripheral vision and can serve to guide search to a particular target scene. Segmenting a multiplex into regions of shared semantic identities also mirrors organisation within single scenes—scenes can be divided up into semantically distinct regions, each differentially likely to contain particular objects (Greene 2013), and this guides search within scenes (Ehinger et al. 2009; Torralba et al. 2006). Thus, providing regions of a multiplex with consistent and peripherally detectable semantics may aid search of a multiplex in a similar way to how it aids search of a single scene.

Once people are informed about where the groups of scenes occur—as they were in Experiments 2 and 3, and as is likely to be the case in a CCTV control room—it may be that the benefit afforded by grouping scenes comes from this explicit knowledge and memory of this instruction rather than any semantic processing of the scenes in the multiplex. At least three findings from the present study argue against this possibility. First, response times reduced considerably over the course of each experiment and this effect did not vary across the experimental manipulations (as shown by the fact that model fits were not improved by including the interactions between trial number and the other fixed effects in any of the three experiments). Therefore, the trial-wise changes in search times did not depend on whether or not participants were aware of where to find particular sets of scenes. Second, a benefit for grouping by semantics was found in Experiment 1, where participants were naive to the manipulation of multiplex organisation. Thus spontaneous use of semantic grouping in multiplexes can be made, even when participants are not informed that this is how scenes are arranged. Third, the benefit of organising scenes by semantic category was attenuated when there were no gaps between scenes in the multiplex in Experiment 1—an effect akin to difficulties in object recognition when objects are surrounded by other objects (Levi 2008; Pelli and Tillman 2008; Whitney and Levi 2011), or embedded in a scene (Davenport and Potter 2004; Vanmarcke and Wagemans 2016) compared to a visually isolated object. The finding that a visual manipulation of spacing between scenes influences the benefit gained from grouping scenes by category again suggests that it is not simply that participants are using the instruction or memory for where sets of scenes are in the multiplex, but rather are using on-the-fly processing of scenes in peripheral vision at least in part during multiplex search.

In most surveillance tasks, certain types of scenes will be monitored or searched-for more than others due to the non-uniform distribution of crime in space and time (Ratcliffe 2010). In this situation, grouping scenes by semantic category might result in a specific—and, from a policing point of view, problematic—cost when searching for scenes in the rarely monitored categories of scenes. Searching for scenes drawn from categories that were rarely the target in Experiment 3 showed the expected overall cost to search time, requiring longer to find than scenes in the commonly targeted category. Slower search for rarer targets is consistent with previous reports of difficulties when searching for rare targets (Hon and Tan 2013; Laberge and Tweedy 1964; Wolfe 2020; Wolfe et al. 2005) and suggests that operators will be slower to orient to rarely monitored scenes, and may be more likely to miss events in these scenes. However, for the rare targets, there was no difference between organised and disorganised multiplexes suggesting that grouping scenes by semantic category did not introduce a further cost to searching for these scenes. The lack of cost for may be specific to the task used in the present study and may not generalise well to other aspects of surveillance. It may be that while grouping scenes by semantic category does not result in costs for finding rarely monitored scenes during reactive surveillance, costs would be seen for these scenes when proactively monitoring the multiplex. This is because grouping together the least-frequently searched scenes should result in a portion of the multiplex that is rarely searched within and so will rarely be looked at during proactive monitoring, reducing the chances of spontaneously looking at scenes in this portion of the multiplex (Ehinger et al. 2009; Torralba et al. 2006) or getting close enough to one of these scenes to spontaneously detect an event (Clarke et al. 2016). In line with this concern, detection performance for incidents when proactively monitoring CCTV footage was lower when participants spontaneously adopted a strategy of prioritising some scenes over others than when all scenes were monitored equally (Hodgetts et al. 2018). Furthermore, there is a potential risk associated with the knowledge that crimes are non-uniformly distributed in space and time in that operators may use their own biases and stereotypes to decide upon the likely places and types of crime rather than the true underlying distributions. Indeed, this might account for some of the findings in Hodgetts et al. (2018), with those who prioritised certain scenes perhaps prioritising the wrong scenes due to their preconceived expectations about what might happen where and thus missing the critical events. The implications of arranging scenes by category for proactive police surveillance could, therefore, be serious—with crimes in unexpected locations being less likely to be detected when multiplexes are arranged by semantic category. This issue clearly requires empirical investigation in order to evaluate these potential risks.

If the findings are to be used to make recommendations for CCTV control room organisation, then the limitations of the paradigm used in the present study that were outlined in the introduction to this article must be re-considered here. The task was simplified and somewhat removed from the real-world surveillance task in order to provide a paradigm that focuses specifically on the process of searching for a target scene within a multiplex array. In real-world surveillance this forms a part of many tasks in proactive and reactive surveillance, but in all cases is part of a broader task that involves elements not captured by the present paradigm. For example, once located, scenes need to be inspected, but the purpose and nature of inspection will differ greatly depending on the operator’s task; for example, inspecting a scene to check for possible crime will require very different perceptual and cognitive processes from inspecting a scene in order to identify or follow a suspect. Indeed the variation in types of surveillance task that operators engage in may account for some of the differences between prior studies of CCTV surveillance, and between these prior studies and the present one. For example, previous reports of no benefit from arranging scenes logically (Harris et al. 2008; Stedmon et al. 2011) compared to the apparent benefit offered by organising the scenes within the multiplex in the present study may arise from differences in the nature of the task—following suspects (Harris et al. 2008; Stedmon et al. 2011) compared to finding scenes in the present study. Similarly, different measures may reflect different parts of the surveillance process—in the present study the focus was on the time required to search for a specific scene and whether this benefits from grouping scenes within the multiplex, whereas prior studies have focused on the frequency with which events in the surveillance footage are detected (Harris et al. 2008; Hodgetts et al. 2018; Stedmon et al. 2011). Detection performance is a measure that reflects the culmination of a range of perceptual and cognitive processes that occur before during and after a specific scene is oriented to. The present findings are specifically centred around the speed of search for a specific scene and will therefore be most likely to be relevant when speedy selection of a scene is the priority for the operator. This might mean that despite our argument that the paradigm is suitable for aspects of reactive and proactive surveillance, it may be less suitable for proactive surveillance where the task is less centred around rapid orienting to a scene.

From a practical point of view, the findings of the present study can be used to suggest that operators may benefit when searching for scenes if scenes in the multiplex are grouped by semantic category. This benefit is likely to be seen during reactive surveillance, when the operator must orient to a scene called in over the radio, but whether it will also aid operators during proactive surveillance requires future study. The benefit of organising scenes by semantic category is enhanced for scenes in the most frequently searched-for category of scenes, and this benefit comes without any statistical evidence for a cost when searching for rarely searched-for categories of scenes.

In conclusion, the present study found consistent evidence that arranging camera feeds in the CCTV control by semantic category is associated with faster search times for finding scenes within a multiplex of 27 real-world surveillance images. These initial findings offer the basis for possible recommendations for CCTV control room organisation and give rise to questions that can be used to generate future empirical investigations: specifically, whether and how well this arrangement of scenes within the multiplex supports other aspects of common surveillance tasks. The present study, therefore, demonstrates that in the safety-critical environment of the CCTV control room, applying principles derived from theoretical understanding of visual search and scene viewing can aid aspects of the surveillance task; specifically, speeding search for scenes within the multiplex array of camera feeds.

## Significance statement

Using a theoretically driven approach, a novel method for arranging camera feeds in the CCTV control room was proposed and evaluated: arranging scenes into groups that share common semantic properties (common gist). This method was inspired by what is known about sources of information from scenes that can be extracted rapidly, in peripheral vision, and from multiple scenes in parallel and therefore is available to guide search for scenes in a large multiplex array. Arranging scenes into groups on the basis of scene semantics resulted in faster search times for scenes across all three experiments in the present study. When the arrangement was not explicitly explained to participants, this benefit was only found when scenes were also separated by gaps between them, which presumably helped to segment the scenes for peripheral processing. This arrangement method was particularly beneficial for search when targets were drawn from scene categories with unequal frequency, benefiting search for the most common category of targets without clear detriment to search for less common categories of targets. Thus, the present study demonstrates that in the safety-critical environment of the CCTV control room, applying principles derived from theoretical understanding of visual search and scene viewing can aid aspects of the surveillance task; specifically, speeding search for scenes within the multiplex array of camera feeds.

## Figures and Tables

**Fig. 9:**
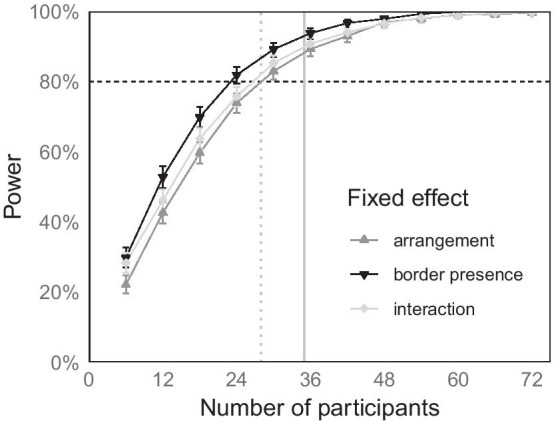
Simulated power using LMM in Experiment 1

**Fig. 10:**
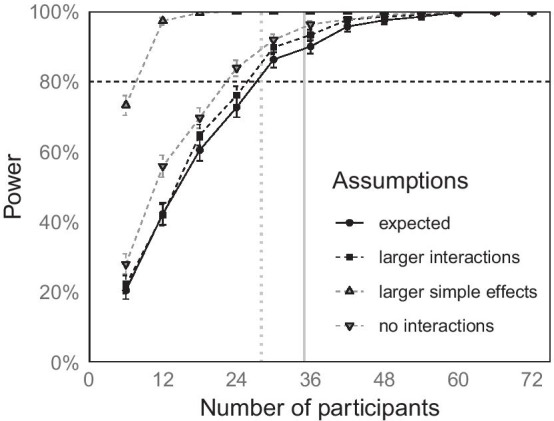
Simulated power for effects of target frequency in Experiment 3

## Data Availability

All data and analysis code from the present study are available on request from Professor Ben Tatler, b.w.tatler@abdn.ac.uk.

## Appendix

See Fig. 9 power was estimated for Experiment 1 by running simulations of the LMM across varying numbers of participants. Points indicate the mean of 1000 simulations, with error bars showing 95% confidence intervals. These power curves were used to justify the sample sizes in Experiments 2 and 3. These curves suggest that a minimum sample size of 28 was required to achieve at least 80% power for all fixed effects in Experiment 2 given the effects found in Experiment 1 (vertical dotted grey line). The vertical solid grey line indicates the number of participants tested in Experiments 1–3.

See Fig. 10. With no prior data on effects of target frequency to base power simulations on, an artificial dataset was created. This dataset reflected the real distribution of target frequency and scene arrangement conditions across each participant. For the simulated LMMs, estimated effects were entered for each fixed effect in the model. For the grand mean, and for effects of arrangement and trial number, these were taken from the LMM run for Experiment 2. For the fixed effects involving target-frequency values were based on the assumption that overall the effects of target frequency were likely to be stronger than those of scene arrangement and also that the direction and magnitude of effect would differ for high-, low- and very low-frequency targets compared to conditions where all categories were equally likely to be the target. Specifically, given an observed effect (beta) of scene arrangement in Experiment 2 of $-0.01$ (faster for categorically arranged multiplexes), we assumed faster responses for high-frequency targets compared to equal frequency targets, with an estimated effect of $-.02$, slightly slower responses for low-frequency targets, with an effect of .01, and even slower responses for very low-frequency targets, with an effect of 0.02. All interactions were estimated to be 0.01 to align with the size of interaction found in Experiment 1 between scene arrangement and border presence. The plot shows the simulated effect of varying participant number on the power for detecting effects of target frequency based on these assumed fixed effects. Alternative assumptions were also explored: (1) there are no interactions between scene arrangement and target frequency, (2) larger interactions between scene arrangement and target frequency (all set to 0.02 rather than 0.01) and (3) larger simple effects of target frequency (all doubled). All of these resulted in more power for any simulated number of participants. The vertical solid grey line in the plot indicates the number of participants analysed (after exclusions) in Experiment 3, and the vertical dotted line indicates the minimum sample size to achieve 80% power for the most conservative of the four assumptions simulated. In the plot, points indicate the mean of 1000 simulations, with error bars showing 95% confidence intervals.
